# Dental caries and its association with the oral microbiomes and HIV in young children—Nigeria (DOMHaIN): a cohort study

**DOI:** 10.1186/s12903-021-01944-y

**Published:** 2021-12-04

**Authors:** Modupe O. Coker, Paul Akhigbe, Esosa Osagie, Nosakhare L. Idemudia, Oghenero Igedegbe, Nneka Chukwumah, Ruxton Adebiyi, Allison E. Mann, Lauren M. O’Connell, Ozo Obuekwe, Augustine Omoigberale, Manhattan E. Charurat, Vincent P. Richards

**Affiliations:** 1 Department of Oral Biology, Rutgers School of Dental Medicine, Rutgers University, 110 Bergen Street, Room C-845, Newark, NJ 07103 USA; 2 Research Department, Institute of Human Virology, Nigeria, Abuja Nigeria; 3 Medical Microbiology Division, Medical Laboratory Services, University of Benin Teaching Hospital, Benin City, Nigeria; 4 Department of Preventive Dentistry, University of Benin, Benin City, Nigeria; 5 Department of Oral and Maxillofacial Surgery, University of Benin, Benin City, Nigeria; 6 Institute of Child Health, University of Benin, Benin City, Nigeria; 7 Institute of Human Virology, University of Maryland School of Medicine, Baltimore, MD USA; 8 Department of Biological Sciences, Clemson University, Clemson, SC USA

**Keywords:** HIV, Caries, Oral microbiome, Cohort, Children, Dental plaque

## Abstract

**Background:**

This study seeks to understand better the mechanisms underlying the increased risk of caries in HIV-infected school-aged Nigerian children by examining the relationship between the plaque microbiome and perinatal HIV infection and exposure. We also seek to investigate how perinatal HIV infection and exposure impact tooth-specific microbiomes' role on caries disease progression.

**Methods:**

The participants in this study were children aged 4 to 11 years recruited from the University of Benin Teaching Hospital (UBTH), Nigeria, between May to November 2019. Overall, 568 children were enrolled in three groups: 189 HIV-infected (HI), 189 HIV-exposed but uninfected (HEU) and 190 HIV-unexposed and uninfected (HUU) as controls at visit 1 with a 2.99% and 4.9﻿0% attrition rate at visit 2 and visit 3 respectively. Data were obtained with standardized questionnaires. Blood samples were collected for HIV, HBV and HCV screening; CD4, CD8 and full blood count analysis; and plasma samples stored for future investigations; oral samples including saliva, buccal swabs, oropharyngeal swab, tongue swab, dental plaque were collected aseptically from participants at different study visits.

**Conclusions:**

Results from the study will provide critical information on how HIV exposure, infection, and treatment, influence the oral microbiome and caries susceptibility in children. By determining the effect on community taxonomic structure and gene expression of dental microbiomes, we will elucidate mechanisms that potentially create a predisposition for developing dental caries. As future plans, the relationship between respiratory tract infections, immune and inflammatory markers with dental caries in perinatal HIV infection and exposure will be investigated.

**Supplementary Information:**

The online version contains supplementary material available at 10.1186/s12903-021-01944-y.

## Background

There is growing evidence of a higher burden, severity, and risk of dental caries with HIV infection [1–10]. However, there is insufficient data explaining the mechanisms underlying this higher risk at the microbial level. While some studies [11–17] have reported significant differences in the bacterial communities comparing lingual and salivary samples in HIV infected (HI) children with uninfected individuals, other studies have observed no significant differences between HI and uninfected children [11–13, 15, 18–22].

The burden of HIV infection is exceptionally high in developing countries in sub-Saharan Africa. Nigeria has the second-highest incidence of HIV infections globally, of which 1.9 million people live with HIV, with 35% of this population being children, and roughly 200,000 children are born to HIV-positive mothers per year [23]. Increased availability and widespread use of highly active antiretroviral therapy (HAART) has led to a significant decrease in the number of children infected with perinatally acquired HIV. However, many of these HIV-exposed-but-uninfected children are believed to have increased chances of developing early-life infections and increased immune system impairment. Early childhood caries (ECC) prevalence is also high in developing countries and can lead to decreased quality of life in children that experience it. Therefore, it is necessary to better understand the impact that HIV status has on the oral bacteriome and mycobiome associated with ECC. Furthermore, with a growing population of HIV exposed but uninfected (HEU) children in sub-Saharan Africa, studies [24–28] have shown an increased risk of early-life infections and mortality, impaired growth as well as a higher (although not significant) risk of dental caries, suggesting perinatal HIV exposure is associated with an immature immune response. Furthermore, previous findings from this population observed that HEU children, like HI children, had lower CD4 values than their unexposed counterparts [25].

To prospectively assess the impact of HIV on oral microbiota and caries risk in children in Nigeria, we initiated the study Dental Caries and its association with Oral Microbiomes and HIV in young children – Nigeria (DOMHaIN—study) in 2019. The DOMHaIN study, which is an ongoing prospective cohort study of young children in Nigeria, aims at providing a detailed taxonomic and functional characterization of dental plaque communities at different stages of caries in HIV-infected (HI), HIV-exposed but uninfected (HEU), and HIV-unexposed and uninfected (HUU) children aged 4 to 11 years in Nigeria by using next-generation sequencing (NGS) technology and tooth-specific sampling. The study aims to characterize the relationship between the plaque microbiome and perinatal HIV infection and exposure and investigate how perinatal HIV infection and exposure impact the microbiome’s role in caries disease progression. Our central hypothesis is that HIV infection and treatment adversely affects the dental microbiome, shifting communities to a state that predisposes a child to caries. Data from this cohort will improve our understanding of the complex relationships between the oral microbiome and health and disease outcomes in HIV- infected children and will contribute to the growing body of literature focused on understanding the human microbiome. Here, we present methods and procedures that were put in place for this unique study in sub-Saharan Africa.  

### Methods/design

#### Study design

DOMHaIN is a prospective cohort study of children aged 4 to 11 years recruited from the University of Benin Teaching Hospital (UBTH) between May and November 2019. Three groups of children were enrolled in this study for comparisons; HIV-infected (HI), HIV-exposed but uninfected (HEU), and HIV-unexposed and uninfected (HUU) as controls.

This study is being implemented in the UBTH, a premier and multi-specialty tertiary healthcare service provider located in Edo State, Southern Nigeria. The prevalence of HIV in this region is the highest in the country at 3.1% among adults aged 15–49 years, with 32% being pregnant women [29]. As a tertiary referral hospital with over 860-bed capacity, UBTH is widely acknowledged as a Centre of Excellence and has remarkably and effectively served as the last port of call for expert management of diverse and varied disease conditions in Edo, Delta, parts of Kogi and Ondo state which primarily forms its catchment area and sometimes further away.

#### Ethical considerations

The institutional review board at the University of Maryland Baltimore (HP-00084081), Rutgers State University of New Jersey (Pro2019002047), and University of Benin Teaching Hospital, Benin City (ADM/E22/A/VOL. VII/14713), Nigeria, approved this study. Study staff took care to verbally explain (in English and Pidgin English) all study activities, and risks and benefits of voluntary participation to parents/guardians or caregivers. Questions were asked to confirm understanding. Written informed consent was then obtained prior to recruitment.

### Recruitment

**Fig. 1:**
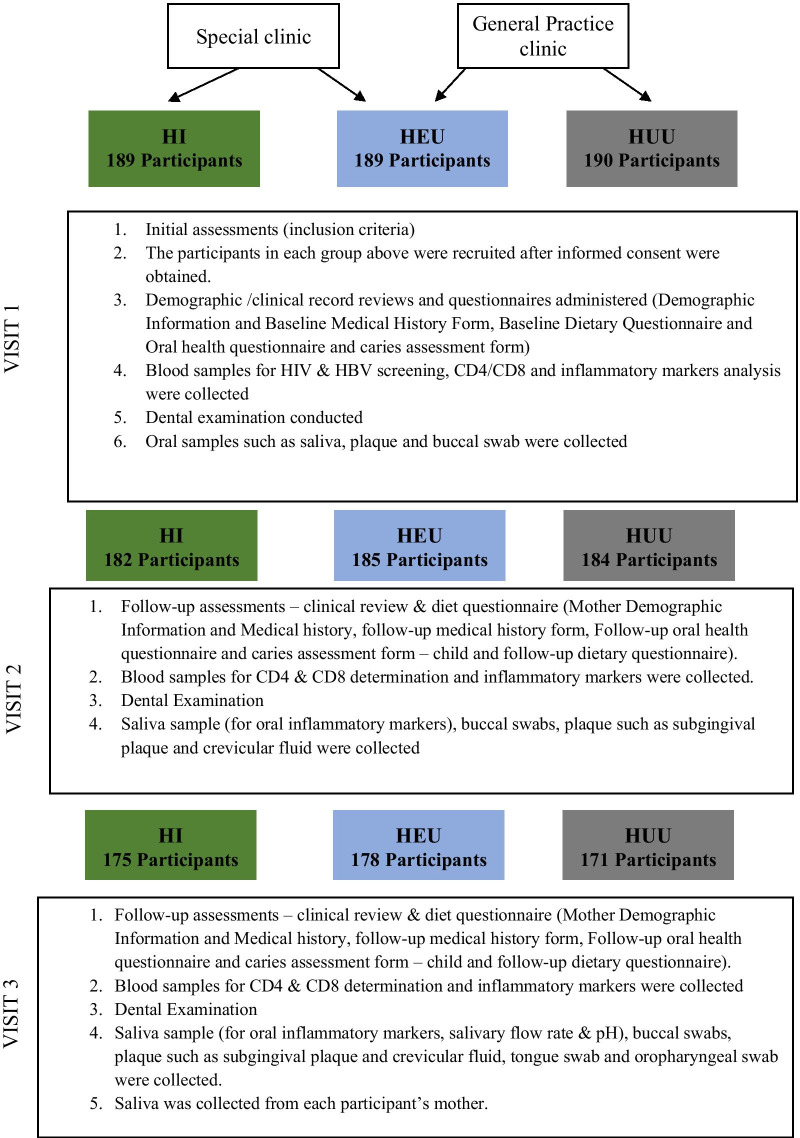
Descriptive flow of recruitment and retention of DOMHaIN participants

The DOMHaIN study is a prospective single-center study with a recruitment process of seven months initiated by approaching the parents of the children between the ages of 4–11 years old attending the pediatrics special treatment HIV clinic for the HI and HEU, and the pediatrics out- patient clinic for the HUU by the trained healthcare professionals. Prior to study initiation, the research team met with representatives of the community, health center staff and leadership within a comprehensive 3-day training workshop. Based on our past experience at this site, this strategy ensures sustained enthusiasm and collaboration and encourages participation in research studies. Once the study began, our principal investigators (PIs) and site leadership organized bi-weekly meetings to address any challenges with recruitment. Community health staff were to informed of the study and provided a simple explanation of the purpose and study procedures. Every guardian and parent who showed a willingness to be engaged in the study were taken through a written and verbal description of the study, in a one-on-one setting, and thereafter signed consent. The trained health professionals attended both clinics mentioned above for the week's five working days to approach eligible participants' mothers. A total of 568 participants, including siblings, were recruited, all of which completed the baseline questionnaire. Figure 1 highlights the participant enrollment procedure. Overall, the 568 children recruited in the study comprised 189 HI and 189 HEU and 190 HUU individuals (Fig. 1). Participating children were tested for HIV to classify them accurately and asked to follow up per the study schedule.

### Follow up

**Fig. 2:**
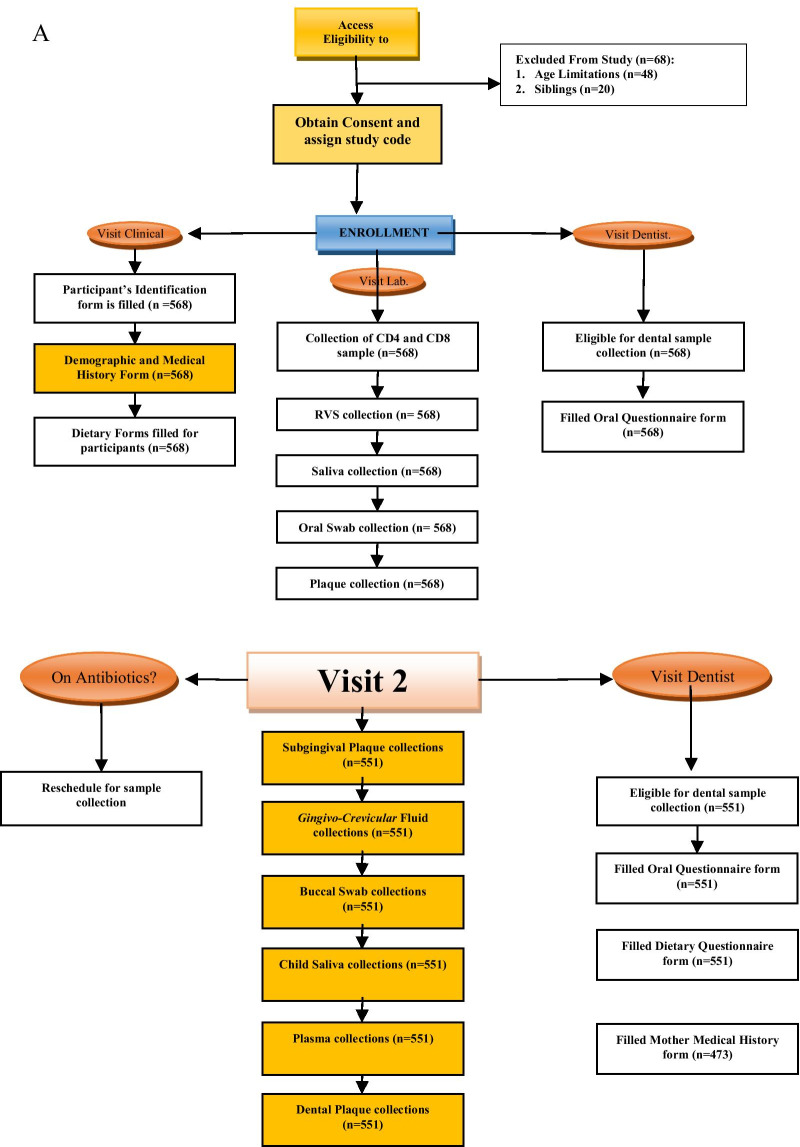

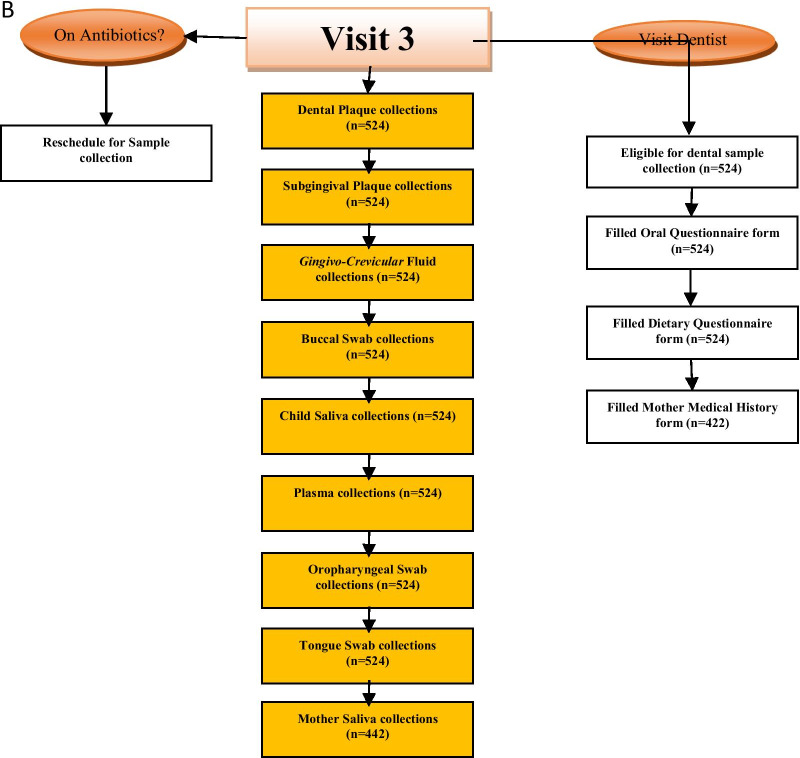
**A** Flow chart of the activities and sample collection at the baseline and first follow-up visits of the DOMHaIN Study. **B** Flow chart of the activities and sample collection at the final follow-up visit of the DOMHaIN Study

The follow-up visits of the study were scheduled into two visits: 6 months’ post-baseline visit (V2), and 12 months’ post-baseline visit (V3). Data were collected through a well-structured questionnaire at every follow-up visit, and sample collections were carried out at all the follow-up visits (Fig. 2a, B). Mothers/caregivers of study participants were contacted using the short message service, voice call, third-party contact, and home visits at one month, two weeks, one week, two days before the visit day, and on the morning of the visit. For participants with academic activities like mid-term tests, exams were rescheduled to a more convenient day within the visit reference period. All participants were regarded as eligible for the follow-up visit except those who voluntarily withdrew from the study, relocated out of the geographical location of the study, deaths or could not be reached by phone/home visit for their previous visit. The samples collected at each visit and the different questionnaires administered are outlined in Fig. 1. The attrition rate at visit 2 and 3 was 3% and 4.9%, respectively, highlighting the success in participant retention despite the COVID-19 pandemic. Recruitment and retention strategies include culturally sensitive, experienced and diverse research team, good communication protocols and use of incentives. Other strategies utilized for this study to meet the needs of the study site and participants, included: collaboration with point of care physicians, responding to the clinical environment; and addressing participants’ health literacy and level of educational attainment of parents/and or care givers. The duration of the scheduled follow-up visits (visit 2 and 3) for all participants in the study was between November 2019 and December 2020.

### Data collection and measures

This study was designed to use data from all available HIV-infected children receiving treatment at the Special Treatment Clinic within the eligible age range. As such, this sample represents a near-complete population of HIV-positive cases for the age group at study initiation. Additionally, this study was planned to be the first longitudinal study to compare oral health status and outcomes in HI, HEU and HUU Nigerian children using the measures outlined below.

### HIV status determination

To accurately identify groups, HIV infection or exposure was determined based on a review of maternal and child medical records and an HIV confirmatory test of the child-participant at the time of enrollment.

Infant HIV testing using HIV DNA PCR was done for all HEU babies at six weeks postpartum and four months for non-breastfed infants or two months after breastfeeding cessation according to the WHO and Nigerian Ministry of Health guidelines on early infant detection (EID) of HIV [30, 31] using the COBAS® AmpliPrep/COBAS® TaqMan® HIV-1 Qualitative Test, version 2.0 (TaqMan® HIV-1 Qual Test version 2.0), a dual-target total nucleic acid real-time PCR assay.

Perinatally exposed or infected children at UBTH initiate ART in line with Nigerian Ministry of Health guidelines, which also recommends either HAART for pregnant women requiring treatment for their disease or option B prophylaxis with triple regimen until one week after breastfeeding ceases, as well as nevirapine to the baby from birth to 6 weeks which informs the time of sample collection (dried blood spots) for EID.

HIV status for HEU children had been previously determined from blood samples collected at 18 months postpartum using rapid antibody tests according to WHO guidelines and Nigeria Ministry of Health approved HIV testing algorithms [30, 31]. For the DOMHaIN study, HEU and HUU children were recruited from the well-baby and child pediatric clinics and their HIV status confirmed with rapid antibody tests.

### Study visits and data collection

Participants were assessed at baseline, six months, and 12 months thereafter. Medical and dental history, demographic data, baseline dietary information and oral health/caries assessment were obtained with the aid of a well-structured questionnaire at all visits. CD4 + T-cell count and percentage values together with CD8 + T-cell count were obtained via flow cytometry. Caregivers were interviewed using standardized questionnaires for the child’s sociodemographic characteristics, feeding, and oral hygiene practices. Maternal and infant medical records, questionnaires, and oral examinations were analyzed with R v3.6.0 (http://www.R-project.org) to analyze the different factors and possibilities that may contribute to the prevalence of caries in HI, HEU, and HU children in Nigeria. Medical history was obtained from these interviews and confirmed or resolved by chart review. CD4 + T-cell counts and percentages were assessed based on the CDC’s 2014 case definitions for stages of HIV infection. Birth weight, current weight, height, and medication use were documented from medical records. Maternal education and employment status were also assessed. Participants that agreed to participate in the study came in for three visits; baseline, six months (V2), and 12 months (V3). Each clinic visit’s component is detailed in Fig. 2a, b.

Standardized questionnaires were used at each study visit to document general medical information, including medication and comorbidity history, general physical examination findings, and anthropometric assessment. Demographic information and personal characteristics were assessed via self-report questionnaires. Questionnaires collected data on demographic factors, medical and oral health history, dental hygiene, dietary intake (via the Food Frequency Questionnaire), and medications.

### Anthropometric measures

All measures were conducted by trained and certified staff according to standardized protocols described below. Height and weight were measured by a trained technician using soft metric tape and a weight scale. During weight measurement, the children were asked and watched not to lean forward or hold any form of support; this was done to make the measurement precise [32]. In addition, the weighing scale was regularly checked by a known weight to ensure its accuracy. During height measurement, the children were asked to stand without footwear, heels together, stand straight and look straight for the Frankfort plane to be parallel to the floor. Measurement was done with the horizontal support on the highest point of the vertex of the head.

### Feeding practices/dietary assessment

Information on the feeding practices of the children were collected with the aid of the baseline and follow-up dietary questionnaires filled by the parent or caregivers of the children at the respective visits, as shown in Table 1. The questionnaires contain information on breastfeeding, including the age of commencement and cessation of breast milk and specific liquid food intake, including details on frequency and number of times in the last 24 h before the date of such visit. Information on the age of introduction to various solid foods and beverages was collected at all applicable visits (Table 1).

**Table 1:** Summary of questions within DOMHAIN Dietary questionnaire from V1 (baseline) to V3 (12-month post-baseline visit). Data collected from the DOMHAIN Dietary questionnaire from V1 (baseline) to V3 (12 months’ post baseline visit)

S/N	Question	V1 Baseline	V2 6 months post baseline	V3 12 months post baseline
1	Have you ever breast fed this child?	*		
2	How soon after birth did you first put your baby to the breast?	*		
3	Did you give your baby colostrum?	*		
4	Did you give your baby water or honey or sugar water before initiating breastfeeding?	*		
5	Did you give your baby any animal milk or infant formula before initiating breastfeeding?	*		
6	At that time, did your child use a bottle or sippy cup to drink fluids other than water?	*		
7	How old was your baby when you stopped breastfeeding?	*		
8	Why did you not breast feed the child?	*		
9	What did you decide to give your baby?	*		
10	At that time, did your child use a bottle or sippy cup to drink fluids other than water?	*		
11	Did your child ever sleep off during breast feeding or bottle feeding?	*		
12	How often did this happen?	*		
13	Does your child ever sleep off during eating solid foods?	*		
14	How often does this happen?	*		
15	Does your child still drink with a bottle?	*		
	List of Food (Questions 16–18 were asked against specific foods).*Check the full list of food items below*			
16	how often?	*	*	*
17	How old was the baby when this liquid or food was first given?	*		
18	How many times in the last 24 h did you give your child this food?	*	*	*

Full list of dietary food for questionnaire

Liquid foods: Tinned or powdered milk, Soya milk, Fura, Pap/Ogi/Akamu, Tea/Chocolate drink, Juices, Sugar or glucose water, Sugar-salt soln (ORS), Antibiotics syrup, Multivitamins syrup, Fizzy drinks e.g. coca cola (mineral/soda), Sugary liquids

Solid Foods: Bread, Rice, Beans, Eba/Amala/Fufu, Maize/other corn meal, Meat/Fish/Chicken, Eggs, Yam/yam pottage, Vegetables/fruits, IndomieNoddles, Cake/Buns/Puff-buff, Sweets/Chocolate, Biscuits/Cookies, Ice-cream, Other solids

### Dental examination and assessment

Dental examination was carried out by qualified, trained, and calibrated dentists using WHO criteria for the assessment of caries [33] and International Caries Detection and Assessment—ICDAS scores [34] who were blinded to the status of the children. The examination was done in a dental chair with a dental mirror, probe, and artificial light. Teeth were classified as caries-free or caries affected with the location of the lesion in the enamel or dentine stated using FDI nomenclature.

### Oral examination

The oral examination involved inspecting the head, neck, and intraoral assessment of the hard and soft tissues was done consistently and uniformly. The physical examination involved the identification of possible lesions, facial asymmetry, and swelling. Palpation of the head and neck was done to identify possible lymphadenopathy, mass and tenderness. The TMJ was examined for any tenderness, and assessed for any limited opening, deviations or any asymmetries. Intraoral examination was performed to determine the presence or absence of caries and their severity using the WHO criteria for the assessment of caries [33] and International Caries Detection and Assessment—ICDAS scores [34]. In addition, the oral hygiene status was also determined.

The Oral Hygiene Index (Simplified)-OHI-S, as proposed by Greene and Vermillion [35, 36] was used to assess the oral hygiene status in this study. OHI-S comprises the Debris and Calculus indices, which were obtained by determining the amount of debris or calculus with regards to 6 numerical determinations found on the surfaces of the index teeth. 11, 16, 26, 31, 36 and 46 and 51, 55, 65, 71, 75, 85 in the permanent and deciduous dentitions respectively [37]. As previously reported, the debris and calculus scores were totaled and divided by the number of surfaces scored for each study subject [37]. The grading scores were good oral hygiene (0–1.2), fair oral hygiene (1.3–3.0), while poor hygiene was designated as scores > 3.1.

### Caries assessment

Caries prevalence and severity were defined using dmft/DMFT for primary/permanent dentition (decayed tooth, missing tooth due to caries, and filled tooth). Radiographs were not used to determine caries definitively or determine the affectation of the pulp. Dentists were trained to examine, calibrate, and effectively diagnose and differentiate between sound surfaces and non-cavitated caries lesions per International Caries Detection and Assessment—ICDAS scores [34] and the WHO criteria for the assessment of caries [33].

To account for variation within microbial communities over time for the same individual, children were followed over a period of 12 months and sampled at baseline (V1), six months (V2), and 12 months (V3). We excluded children that: (i) have been treated with antibiotics/antifungals within the past three months, and (ii) have orthodontic appliances. Plaque samples are being characterized according to the condition of tooth of origin as follows: (i) tooth surface that is caries-free (PF; ICDAS = 0), (ii) tooth surface that has an active, enamel carious lesion (PE; ICDAS = 1–3), and (iii) tooth surface that has an active, dentin carious lesion (PD; ICDAS ≥ 4) [38]. Second, children were grouped by caries status: (i) caries-free (CF) with no clinical or reported evidence of caries experience [decayed, missing and filled teeth (DMFT) = 0]; (ii) caries-active with enamel lesions only [(CAE); DT = 0; MFT ≥ 0]; and (iii) caries-active with at least two cavitated, unrestored dentin carious lesions [(CA); DT ≥ 2, MFT ≥ 0]. Then, considering the caries status of the child, each plaque sample will be placed in one of six categories as follows: (1) caries free child; sample obtained from a caries free tooth surface (CF-PF), (2) child with active, enamel carious lesions; sample obtained from a caries free tooth surface (CAE-PF), (3) child with active, enamel carious lesions; sample obtained from an active, enamel carious lesion (CAE-PE), (4) child with at least two cavitated, unrestored dentin carious lesions; sample obtained from a caries free tooth surface (CA-PF), (5) child with at least two cavitated, unrestored dentin carious lesions; sample obtained from an active, enamel carious lesion (CA-PE), and (6) child with at least two cavitated, unrestored dentin carious lesions; sample obtained from an active dentin carious lesion (CA-PD). Lastly, children were categorized according to their HIV status: (1) HI children receiving HAART (2) HIV exposed but uninfected children (HEU), and unexposed and uninfected (HUU) children. For any child, it was possible to collect up to three different categories of plaque sample (based on the condition of the tooth) (PF, PE, PD). Due to differences in caries status among children, we estimated an average of two plaque samples per child will be collected. This equates to 200 plaque samples per HIV status with 50 for each of the six overall plaque categories. Plaque samples were stored in RNA*later* (Qiagen) at −80 °C. Twice a year, through years 1 to 3, samples were shipped frozen on dry ice directly from UBTH/IHVN in Nigeria to the PI’s laboratory at Clemson (Richards) or Rutgers (Coker).

### Sample collection

Oral samples such as saliva, buccal swabs, gingivo-crevicular fluid, oropharyngeal swab, supragingival plaque, subgingival plaque, and tongue swab samples were also collected aseptically from each participant, appropriately labelled and stored at − 80 °C (plaque, and saliva) or at − 20 °C (buccal swab and tongue swab) for high throughput next-generation sequencing to provide a detailed taxonomic and functional characterization of the oral microbiome.

Pre-collection preparation—All participants were requested to refrain from food for 2 h and oral hygiene (brushing or flossing the teeth) for 12 h prior to sample collection. The supragingival plaque was collected at the lingual aspect of selected mandibular anterior teeth and the buccal part of selected maxillary posterior teeth using a sterile Gracey’s curette®. The plaque scrapings were placed into a sterile, pre-labelled, 2 ml cryogenic vials containing 500 ml of RNA later, put on ice immediately after sampling, then transported to the laboratory within 2 h of collection for storage at − 80 °C in a 9 × 9 laminated cardboard storage boxes. Sub-gingival plaque samples were collected from the mesio-buccal sites of first molars, from the apical extent of the periodontal pocket or gingival crevice and drawing it coronally with slight pressure. However, if there is a diseased tooth or mucosa, subgingival plaque will be collected from that site and documented as such. The subgingival plaque was collected using a sterile Gracey’s curette® and then placed immediately into a sterile conical tube. The tube was sealed and labeled with computer-generated labels. Samples were kept cool after collection by storing the samples over ice. Samples were stored and frozen at − 80 °C with 9 × 9 laminated cardboard storage. Samples were obtained in the morning (before noon), put on ice immediately after sampling, transported to the laboratory within 2 h and stored at − 80 °C before further processing [39, 40]. Based on tooth health, up to three types of plaque samples were obtained from each participant in a site (individual tooth) specific manner. Individual samples from separate teeth were not pooled (PF = tooth surface that is caries-free (ICDAS = 0), PE = tooth surface that has an active, enamel carious lesion (ICDAS = 1–3); PD = tooth surface that has a vibrant, dentin carious lesion (ICDAS ≥ 4).

Gingivo-crevicular fluid (GCF) samples were collected with the aid of Mynol® absolute paper point and periopaper strip®, respectively. The participants were asked to open their mouth wide and their tongues were suspended with the aid of a wooden spatula. The sterile paper point and periopaper strip were picked with sterilized toothed college tweezers and placed at the surface of the molar teeth and incisor to absorb fluid for about one minute and afterwards transferred to the pre-labelled 2 ml cryogenic tube on ice. As for supra- and sub-gingival plaque collection, samples were transported on ice after collection to the laboratory and subsequently stored at − 80 °C in 9 × 9 laminated cardboard storage boxes.

The buccal swab was collected with Isohelix Sk-2® swab stick. The swab stick rubbed against the buccal wall of the mouth ~50 times for 30 s and placed in a pre-labelled 5 ml cryovials with Isohelix® Dri-capsules and placed on ice. After collection, samples were transported on ice to the lab and were properly labelled and stored with a 7 × 7 laminated cardboard storage box at-20^0^C for high throughput next-generation sequencing to provide a detailed taxonomic and gene expression characterization of the oral microbiome.

Unstimulated child saliva samples were collected; the participant was asked to lie on the examination couch with the head raised up. With the aid of a wooden spatula to suspend the upper and the lower teeth, saliva was allowed to gather in the mouth for about five minutes and thereafter aspirated with the aid of a sterile plastic pasture pipette into the two separate pre-labelled 2 ml cryogenic vials on ice. After collection, samples were transported on ice to the lab and were properly labelled and stored at −80 °C in a 9 × 9 laminated cardboard storage box. Unstimulated saliva samples were also collected from mothers (where available); these adult participants were given a wide mouth 50 ml falcon tube, and participants were asked to produce 5 ml saliva by drooling into the falcon tube. Samples were aliquoted into two 2 ml pre-labelled cryogenic vials on ice. After collection, samples were transported on ice to the lab and were properly labelled and stored at − 80 °C in a 9 × 9 laminated cardboard storage box.

Tongue swabs were also collected with Isohelix Sk-2® swab kits, with the aid of a wooden spatula to suspend the upper and the lower teeth. Similar to buccal swabs, swab sticks were rubbed against the tongue’s surface 50 times for 30 s and placed in pre-labelled 5 ml cryogenic vials with Dri-capsules and placed on ice. Cold chain was maintained from the point of collection to the laboratory, where it was processed and stored at −20 °C in a 7 × 7 laminated cardboard storage box.

The oropharyngeal swabs were collected by a trained dentist positioned by the side of the participants. The participant’s head was tilted to the back slightly and asked to open their mouth wide enough to expose the tonsils and back of the throat. A tongue depressor was used to hold the tongue away from the back of the throat to expose the area of redness and white spots on the tonsils. The oropharyngeal swab was rubbed over the area identified without touching the tongue and throat. The head of the swab was broken off and placed into pre-labelled 5 ml cryogenic vials on ice containing 1 ml RNAlater. After collection, samples were transported on ice to the lab and were properly labelled and stored at −80 °C in a 7 × 7 laminated cardboard storage box. Note for all samples collected, cold chain was maintained from the point of collection to the laboratory where it was processed and stored [40].

Shipment of samples from the collection site to Clemson University, USA, was according to the International Air Transport Association (IATA) standard of practice and scheduled approximately every six months. Samples were transported on dry ice (solidified carbon (IV) oxide) in 7 × 7 laminated cardboard storage boxes for all swabs samples and 9 × 9 laminated cardboard storage boxes for plaque and saliva samples in SAF-T absorbent and transparent biohazard bags with the services of an international courier.

### pH determination

The saliva pH of each participant was taken with the aid of MColourpHast™ (pH-indicator strips (non-bleeding) pH 0–14 universal indicator). Saliva was collected as stated above, and the four colours shade embedded pH 0–14 deep strip was dipped into the sample and timed for 2 min with the aid of a timer. The colour change on the pH strip was matched at the end of the 2 min with the colour meter on the strip pack ranging from 0 to 14, and the value of the colour match was recorded accordingly.

### Salivary flow rate determination

Participants were asked to lie on the dental chair. With the aid of a wooden spatula to suspend the upper and the lower teeth, whole unstimulated saliva was allowed to gather in the mouth for about five minutes and thereafter aspirated with the aid of sterile plastic pasture pipette into a graduated 15 ml Falcon tube, and the volume measured and recorded. The recorded value was divided by the time of saliva production (usually 5 min); values were recorded as salivary flow rate.

### Blood measures

10 ml of whole blood was obtained from each participant by venous puncture with vacuum pressure method of blood collection and dispensed in two aliquots of 5 ml each in EDTA containers with one of the aliquots used for retroviral, HBV and HCV screening and plasma stored for future IL-10, IL-2, IL-6 and C-reactive protein assays while the second aliquot was used for CD4 and CD8 flow cytometric analysis.

### Determination of HIV status with serological HIV testing kits

The determination of the HIV status of the perinatal HIV exposed children in this study was in accordance with the Nigeria's Federal Ministry of Health HIV Care and Treatment Guidelines. Early infant diagnosis was performed at four months for children born to non-breastfeeding mothers and two months after cessation of breastfeeding for children of  breastfeeding mothers and results were retrieved from the patient’s folder. The serological kit of the national algorithm was used in screening the exposed-uninfected (HEU) and the unexposed uninfected (HUU) children in the study to reaffirm their status, such as the Determine kit as the first line of kit, Unigold kit to confirm the positive result from Determine and Statpak as the tiebreaker for all indeterminate outcomes from the former kits (Determine and Unigold) [41] (See Supplementary Information-Additional File 1).

### CD4 and CD8 cell count estimations were done using flow cytometry

*Principle of the test.* Flow cytometry is a method of differentiating cells or micro-particles in suspension and counted according to the cell size and internal structure. In the Sysmex/Partec Cyflow Counter II, the fluorescent monoclonal antibodies bind to the CD4 or CD8 antigen on the mononuclear cell (T-lymphocytes and monocytes). In a buffer suspension, the complex is passed through the flow cuvette in a single stream of flow. The complex is excited by the solid-state laser light at a wavelength of 532 nm, causing the complex to emit light captured by a photomultiplier tube and transmitted into digital readout as count [42].

*Procedure:* 20 ml of CD4 or CD8 monoclonal antibody was introduced into a Rohren test tube, and 20 ml of well mixed whole blood collected within 6 h was added. It was mixed and incubated in the dark for 15 min at room temperature. 800 µl of no-lyse buffer were added, mixed, and read on the Sysmex/Partec Cyflow counter II. The prepared sample was plugged into the port of the Cyflow and allowed for acquisition and data analysis. The Cyflow starts from pre-run, run, count & stop. The Cyflow counts a known volume of the sample and stops. The Cyflow operation is an actual volumetric absolute counting. It counts only 0.2mls of the prepared sample. For CD4 or CD8 T-lymphocyte absolute count, the important value is the count/µl of blood obtained. The result was calculated as follows:

$$Cells \;per\; microliter = \frac{n \times Dilution\; factor}{{1000}}$$

where n = count/µl; Dilution factor = 42.

### Viral load determination using real-time polymerase chain reaction (RT-PCR)

*Procedure A Sample and Control Preparation:* 1050 µl of each specimen was vortexed and transferred with the control to the input -5 tube using a micropipette [43]. All specimens and control were transferred to the sample/control rack starting with high (+), low (+), negative (−), then followed by the samples. The sample/control rack was loaded into the instrument.

### Map (magnetic glass particle)

The procedure involved three stages: lysis, stabilization, and deproteination—the addition of lysis buffer results in the complete lysis of the sample. DNA and RNA are released and simultaneously stabilized with the degradation of inhibitory proteins and RNase by protease digestion and inactivation of nucleases by chaotropic salt, reducing agent and detergent. Total nucleic acids released by lysing buffer bind to the silica surface of added magnetic glass particles (MGP) through capturing. Wash buffer was added to remove unbound substances and allow impurities like denatured protein, cellular debris, potential PCR inhibitors. The purified total nucleic acids are released at elevated temperature (80 °C), high pH conditions and low salt concentration through the elution process, and all these activities were carried out in a sterile class II biosafety cabinet. The k-tubes are then transferred to the COBAS Taqman for amplification. The results are read in the Amplink as copies/ml [43]. Blood samples were drawn by a trained technician using venipuncture and then centrifuged, portioned to plasma and then stored in − 80 °C freezers in 0.5 ml aliquots. The additional sample remains for future use in the bio-specimen bank.

### DNA/RNA extraction of supragingival plaque

Upon arrival to Clemson, SC, USA, the plaque samples were checked to ensure that they were still frozen. After verifying that the samples were frozen, they were stored at −80 °C. A 150 µl aliquot of the supragingival plaque samples were used for the DNA extraction protocol. The DNA was extracted using the Qiagen DNeasyPowerBiofilm Kit (Qiagen, USA) according to manufacturer’s protocol. An extraction blank was included in each set of extractions to monitor for external contamination. After successful extraction, the concentration of the genomic DNA for each extracted sample was measured using Qubit 3.0 (Thermo Fisher, USA).

### Next-generation sequencing

To characterize the full bacterial community of both saliva and dental plaque samples, we used a gene-fragment meta-barcoding approach targeting the V4 hypervariable region of the 16S rRNA gene using previously published primers [44, 45]. As 16S rRNA amplicon sequencing can only generally resolve taxa to the genus level (with varying resolution among taxonomic groups) [46], we developed two sets of primers that target a fragment of the 30S-S11 and 50S-L6 rRNA genes to characterize strain-level diversity of two important oral groups, *Streptococcus* spp. and *Neisseria* spp., respectively. In addition to the bacterial community, we generated data on the oral fungal community using fungal-specific primers that target the ITS2 region of the eukaryotic rDNA complex [47]. Each dataset was built into Illumina libraries and sequenced on a MiSeq using paired-end 2 × 250nt reads. We first trimmed primer and adapter sequences from the raw fastq files using Cutadapt [48]. Next, we quality-filtered, merged paired-end reads, removed chimeric sequences, and generated amplicon sequencing variants (ASVs) from our trimmed reads using the DADA2 pipeline [49]. Finally, we assigned taxonomy to each ASV using VSEARCH [50] using the bacterial EzBiocloud [45] or fungal UNITE [51] databases as a reference. Diversity analyses were performed primarily within the R environment [52] using PhILR [53], Vegan [54], and Phyloseq [55], depending on the length of the amplicon.

### Data processing and quality control

**Fig. 3:**
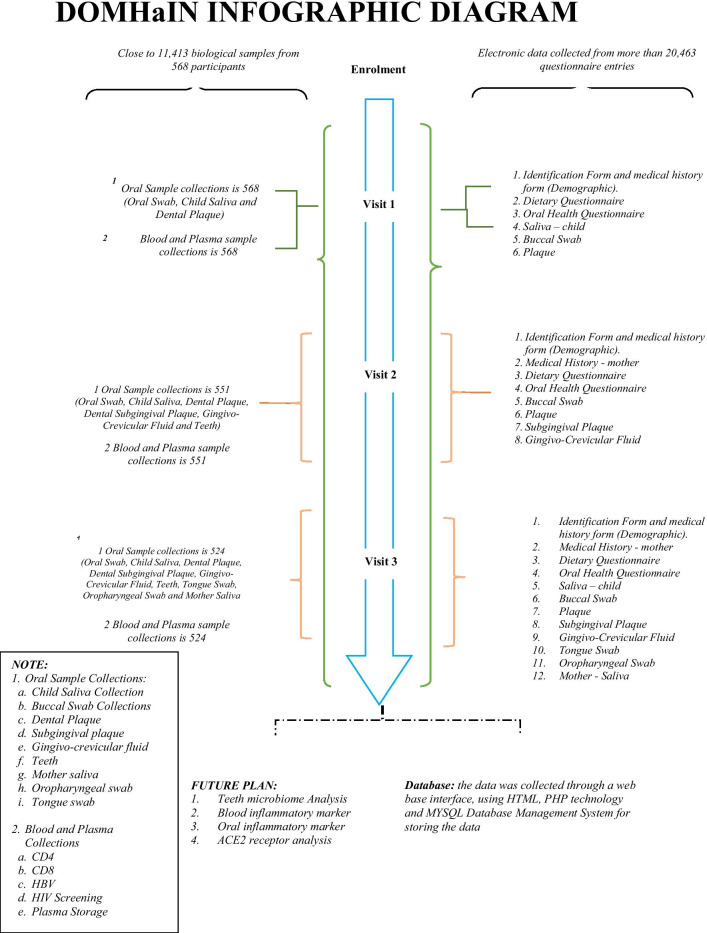
Data repository of the different visits of the DOMHaIN Study

After each study visit, research staff reviewed the accuracy and completeness of hard-copy study forms. Hard-copy forms were scanned and entered electronically into a data management system. Quality-of-life (QoL) self-report data was transferred to REDCap. On a regular basis, the database manager, study coordinator and PIs reviewed the data for inaccuracies and discrepancies. The study has had a successful implementation of its significant components. Figure 3 gives an overview of the data repository for the study.

Biological samples are accurately labelled before processing and/or storage sites at the −80 °C freezers within the University of Benin Teaching Hospital (UBTH). Quality-control checks were performed on all saliva, buccal swabs and plaque samples. Samples were shipped under dry ice to Clemson University for processing/sequencing) and Rutgers University (for biobanking). The PI (Coker) visited the sites at the beginning of each visit cycle and conducted trainings to ensure clinical and dental parameters are collected with the highest quality. In addition, there were biweekly conference calls between the investigators and research staff to address any concerns related to study recruitment, logistics or management.

### Calibration

Calibration sessions for caries identification and classification occurred prior to the initiation of each study (baseline, visit 2 and visit 3). In calibrating examiner-dentists, inter- and intra-examiner reliability rates were determined. Examining dentists were trained to calibrate accurate DMFT (decayed, missing, filled tooth) charting. Inter-rater reliability was determined by comparing assessments performed by each examiner-dentist against the assessment from a gold standard examiner. Intra-rater reliability was determined by comparing assessments performed by each dentist-examiner on the same participant. At each calibration session, screenings were performed on two children with caries.

*Examiner Calibration.* To reduce inter-examiner data collection inconsistency, including carious lesions classification and recording of existing restorations, the study examining dentists were trained to calibrate accurate DMFT (decayed, missing, filled tooth) charting.

The calibration of the three examiners (dentists) who were blinded on the participants’ groups (HI, HEU and HUU) was carried out during three calibration sessions (V1, V2 and V3) between 2019 and 2020. Kappa statistics were used to determine independent inter-examiner reliability on 22 pilot participants in three sessions.

### Power

In previous 16S amplicon sequencing work (PI—Richards) with caries active children of a similar age range [56], we sequenced 185 samples using v2 chemistry in a single MiSeq run. This produced an average of 64,938 reads/sample. Based on six sample categories described above (CF-PF, CAE-PF, CAE-PE, CA-PF, CA-PE, and CA-PD) and given a similar sequencing depth (192 samples in each MiSeq run), a simulation-based power analysis estimated that 50 samples/category will result in overall power in excess of 0.99. In this power analysis, a total of 1,000 simulated data sets were randomly drawn from an infinite population with a multivariate distribution identical to the one in the previous study on caries active children of a similar age range. For each simulated data set, a permutational multivariate analysis of variance (PERMANOVA) test was run at the 5% level using 1,000 random permutations and utilizing the Bray–Curtis dissimilarity index via the adonis function in the R package vegan [57].

## Discussion

The main objective of this cohort is to characterize the oral bacterial and fungal microbiomes using site-specific sampling of different teeth based on six specific caries disease-states in children born to HIV infected mothers. In this study, parents/caregivers of eligible children were invited to participate. This report describes the recruitment strategies and study protocol. Results from the DOMHaIN study would support the microbial link between HIV infection and caries. We will be able to examine the role of salivary flow and pH in caries risk among HIV-infected children. By examining longitudinal measures of caries in DOMHaIN study, we would be to investigate risk factors associated with caries. We also intend to explore the relationship between immune and inflammatory markers with dental caries in perinatal HIV infection and exposure.

The DOMHaIN cohort fills a significant gap in understanding the relationship between the oral microbiome and disease outcomes. The prospective study design, cohort participant size, and linkage with a birth cohort study titled Microbiome Affects the Growth and development of HIV exposed children in Nigeria (MARGIN) are vital strengths. To the best of our knowledge, the DOMHaIN study is the first study able to answer questions about how the oral microbiome changes as children age, determine the relationship of the oral microbiome composition and caries initiation and progression while exploring the association of the oral microbiome with childhood outcomes such as obesity, heart diseases, tuberculosis and periodontal disease. This prospective microbiota-focused cohort of school-aged children enables us to study the individual determinants, rate and patterns of microbiota colonization and succession at the mixed dentition phase. The use of novel metagenomics technology is another strength as the data generated from this cohort lie at the intersection of epidemiology, microbiology, bioinformatics, and biostatistics. When complete, the data can be used to answer an extensive range of questions about the oral microbiome and HIV disease progression in young children.

The main limitation of the cohort is that we would not be able to isolate the effect of ART on caries incidence as HI children at UBTH are placed on antiretroviral therapy immediately after they were enrolled in the HIV care programs as per national guidelines. This may affect the study findings because ART impacts the oral microbiota and general oral health [58, 59]. Furthermore, most HI children received similar medications so, we are not powered to examine the impact of specific ART regimens on the oral microbiota. While there will be no radiological investigation to objectively assess caries or carious lesions involving the pulp, dental examiners were well trained and calibrated. To mitigate these weaknesses to some extent, we will be able to compare some our findings to other cohorts in Nigeria, and we are developing collaborations with other international cohorts.

We also learned valuable lessons applicable to researchers interested in microbiome research in sub-Saharan Africa. Most useful, perhaps, is the importance of collaborating with investigators across disciplines when assembling cohort studies for human microbiome research, development of protocol for the extraction of DNA and RNA from sample collected from specialized sites like the oral cavity and handling of samples for microbiome analysis. Collaboration between researchers with diverse areas of expertise (epidemiology, dentistry/oral biology, statistics, biochemistry, and bioinformatics) has enabled this study to succeed.

To our knowledge, no study has prospectively and comprehensively examined tooth-specific microbiomes in the context of HIV infection, exposure and treatment in young children. The DOMHaIN protocol is designed to examine early-life factors, including perinatal HIV infection and exposure, hypothesized to contribute to dental caries. The proposed research will contribute to our knowledge regarding disease progression in the context of HIV prevention and treatment by comprehensively studying six progressive stages of caries as well as integrating fungal and bacterial transcriptomics. Through DOMHaIN Study, a more complete understanding of the mechanisms underlying the different stages of caries progression in HIV infected children will provide insight into the development of novel preventive and therapeutic interventions.

### Collaboration goals

We intend that several collaborations will enable us to address our study aims, but we also welcome further propositions. We can handle a broad range of research questions with a wide range of different oral and blood samples and detailed clinical and dietary information at our disposal. Therefore, intending collaborators should reach out to the corresponding author of this cohort study for further details.

## Supplementary Information

**Additional file 1**. Supplementary Methods for HIV testing and status determination: Study Procedures.

## Data Availability

All metagenomic and metatranscriptomic sequence data will be made publicly available within National Center for Biotechnology Information (NCBI)’s Sequence Read Archive (SRA) database upon publication of the results in scientific articles. Due to ethical reasons, questionnaires and clinical data cannot be made freely accessible even in de-identified form without an application process that includes submitting a research plan that will first undergo evaluation by the study’s scientific board and then by relevant research ethics committees.

## Abbreviations

16S: 16 sediment coefficient component of ribosomal small subunit
ART: Antiretroviral therapy
CA: Caries active in dentin
CAE: Caries active in enamel
CDC: Centers for Disease Control
CF: Caries-free
DNA: Deoxyribonucleic acid
DOMHaIN: Dental Caries and its association with Oral Microbiomes and HIV in young children—Nigeria
ECC: Early Childhood Caries
HAART: Highly Active Antiretroviral Treatment
HEU: HIV-exposed-uninfected
HI: HI-infected
HIV: Human Immunodeficiency Virus
HUU: HIV-unexposed-uninfected
ICDAS: International Caries Detection and Assessment
ITS: Internal Transcribed Spacer
PE: Plaque from tooth that is identified as being caries-active in enamel
PD: Plaque from tooth that is identified as being caries-active in dentine
PI: Principal Investigator
RNA: Ribonucleic acid
rRNA: Ribosomal RNA
RT-PCR: Real-Time Polymerase Chain Reaction
TMJ: Tempomandibular joint
WHO: World Health Organisation

## References

[1] Rajonson N (2017). High prevalence of dental caries among HIV-infected children in West Africa compared to uninfected siblings. J Public Health Dent.

[2] Coker M (2018). Perinatal HIV infection and exposure and their association with dental caries in Nigerian Children. Pediatr Infect Dis J.

[3] Beena JP (2011). Prevalence of dental caries and its correlation with the immunologic profile in HIV-Infected children on antiretroviral therapy. Eur J Paediatr Dent.

[4] Joosab Z, Yengopal V, Nqcobo CB (2012). Caries prevalence among HIV-infected children between four and ten years old at a paediatric virology out-patients ward in Johannesburg, Gauteng Province, South Africa. SADJ.

[5] Obileye MF (2009). Dental caries status of HIV infected children in Nigeria. Nig Q J Hosp Med.

[6] Tofsky N (2000). Dental caries in HIV-infected children versus household peers: two-year findings. Pediatr Dent.

[7] Hicks MJ (2000). Dental caries in HIV-infected children: a longitudinal study. Pediatr Dent.

[8] Madigan A (1996). Caries experience and cariogenic markers in HIV-positive children and their siblings. Pediatr Dent.

[9] Valdez IH, Pizzo PA, Atkinson JC (1994). Oral health of pediatric AIDS patients: a hospital-based study. ASDC J Dent Child.

[10] Howell RB (1992). Dental caries in HIV-infected children. Pediatr Dent.

[11] Kistler JO (2015). The oral microbiome in human immunodeficiency virus (HIV)-positive individuals. J Med Microbiol.

[12] Li Y (2014). HIV infection and microbial diversity in saliva. J Clin Microbiol.

[13] Dang AT (2012). Evidence of an increased pathogenic footprint in the lingual microbiome of untreated HIV infected patients. BMC Microbiol.

[14] Beck JM (2015). Multicenter comparison of lung and oral microbiomes of HIV-infected and HIV-uninfected individuals. Am J Respir Crit Care Med.

[15] Goldberg BE, et al. The Oral Bacterial Communities of Children with Well-Controlled HIV Infection and without HIV Infection. PLoS One; 2015. **10**(7): p. e0131615. 10.1371/journal.pone.0131615 PMC4492946 26146997

[16] Mukherjee PK., et al. Oral mycobiome analysis of HIV-infected patients: identification of Pichia as an antagonist of opportunistic fungi. PLoS Pathog; 2014. **10**(3): p. e1003996. 10.1371/journal.ppat.1003996 PMC3953492 24626467

[17] Hegde MC (2014). Oral microflora: a comparative study in hiv and normal patients. Indian J Otolaryngol Head Neck Surg.

[18] Mukherjee PK, et al. Dysbiosis in the oral bacterial and fungal microbiome of HIV-infected subjects is associated with clinical and immunologic variables of HIV infection. PLoS One, 2018. **13**(7): p. e0200285. 10.1371/journal.pone.0200285 PMC6040710 29995962

[19] Moyes DL (2016). The gut and oral microbiome in HIV disease: a workshop report. Oral Dis.

[20] Saxena D (2012). Human microbiome and HIV/AIDS. Curr HIV/AIDS Rep.

[21] Liu G (2012). HIV infection affects Streptococcus mutans levels, but not genotypes. J Dent Res.

[22] Starr JR (2018). Oral microbiota in youth with perinatally acquired HIV infection. Microbiome.

[23] Brennan AT (2016). A meta-analysis assessing all-cause mortality in HIV-exposed uninfected compared with HIV-unexposed uninfected infants and children. AIDS.

[24] Evans C, Jones CE, Prendergast AJ (2016). HIV-exposed, uninfected infants: new global challenges in the era of paediatric HIV elimination. Lancet Infect Dis.

[25] Coker M., et al. Perinatal HIV Infection and Exposure and their Association with Dental Caries in Nigerian Children. Pediatr Infect Dis J. 2017. 10.1097/INF.0000000000001702 PMC5725234 28746260

[26] Jumare J (2019). Compromised growth among HIV-exposed uninfected compared with unexposed children in Nigeria. Pediatr Infect Dis J.

[27] Coker MO (2020). Immune status, and not HIV infection or exposure, drives the development of the oral microbiota. Sci Rep.

[28] le Roux SM, et al. Infectious morbidity of breastfed, HIV-exposed uninfected infants under conditions of universal antiretroviral therapy in South Africa: a prospective cohort study. Lancet Child Adolesc Health, 2020. 10.1016/S2352-4642(19)30375-X PMC7235356 31932246

[29] Federal Ministry of Health, N. Nigeria HIV/AIDS Indicator and Impact Survey (NAIIS) 2018:Technical Report. 2019: Abuja, Nigeria.

[30] Federal Ministry of Health, N. National Guidelines for HIV Prevention Treatment and Care. 2016.

[31] National Agency for the Control of AIDS, N. National Strategic Framework on HIV and AIDS: 2017–2021. 2017.

[32] Poh BK (2012). Nutritional status, dietary intake patterns and nutrition knowledge of children aged 5–6 years attending kindergartens in the Klang Valley, Malaysia Malays. J Nutr.

[33] World Health Organization. Oral health surveys : basic methods, 4th ed. 1997: World Health Organization.

[34] ICDAS Coordinating Committee, I.C.D.a.A.S.I.C.C. Rationale and Evidence for the International Caries Detection and Assessment System (ICDAS II). In Clinical Models Workshop: Remin-Demin, Precavitaion, Caries: proceedings of the 7th Indiana conference. 2005: Indianapolis, USA. p. 161–221.

[35] Rashed MA, Taha SE (1995). Oral hygiene index simplified of high and low socioeconomic levels (9–13 years) school children. Egypt Dent J.

[36] Greene JC, Vermillion JR (1964). The simplified oral hygiene index. J Am Dent Assoc.

[37] Folayan MO (2018). General anxiety, dental anxiety, digit sucking, caries and oral hygiene status of children resident in a semi-urban population in Nigeria. BMC Oral Health.

[38] Nascimento, M.M., et al. Oral arginine metabolism may decrease the risk for dental caries in children. J Dent Res, 2013. 10.1177/0022034513487907 PMC3684231 23640952

[39] do Nascimento, C., et al. Impact of temperature and time storage on the microbial detection of oral samples by Checkerboard DNA-DNA hybridization method. Arch Oral Biol. 2014. **59**(1): p. 12–21. 10.1016/j.archoralbio.2013.10.007 24246268

[40] Katsoulis J (2005). Impact of sample storage on detection of periodontal bacteria. Oral Microbiol Immunol.

[41] Charles TEO. Human immunodeficiency virus testing algorithm in resource limiting settings, in Current perspectives in HIV infection [Internet], S. Saxena, Editor. 2013: InTech.

[42] Fryland M (2006). The Partec CyFlow Counter could provide an option for CD4+ T-cell monitoring in the context of scaling-up antiretroviral treatment at the district level in Malawi. Trans R Soc Trop Med Hyg.

[43] Pas, S., et al. Performance evaluation of the new Roche Cobas AmpliPrep/Cobas TaqMan HIV-1 test version 2.0 for quantification of human immunodeficiency virus type 1 RNA. J Clin Microbiol; 2010. **48**(4): p. 1195–200. 10.1128/JCM.01832-09 PMC2849552 20164281

[44] Teng F (2018). Impact of DNA extraction method and targeted 16S-rRNA hypervariable region on oral microbiota profiling. Sci Rep.

[45] Yoon SH (2017). Introducing EzBioCloud: a taxonomically united database of 16S rRNA gene sequences and whole-genome assemblies. Int J Syst Evol Microbiol.

[46] Johnson JS (2019). Evaluation of 16S rRNA gene sequencing for species and strain-level microbiome analysis. Nat Commun.

[47] Caporaso JG (2011). Global patterns of 16S rRNA diversity at a depth of millions of sequences per sample. Proc Natl Acad Sci USA.

[48] Martin, M. Cutadapt removes adapter sequences from high-throughput sequencing reads. EMBnet. J. 2011; 17, p. 10–12.

[49] Callahan BJ (2016). DADA2: High-resolution sample inference from Illumina amplicon data. Nat Methods.

[50] Rognes, T., et al. VSEARCH: a versatile open source tool for metagenomics. PeerJ, 2016. 4: p. e2584. 10.7717/peerj.2584 PMC5075697 27781170

[51] Nilsson RH (2019). The UNITE database for molecular identification of fungi: handling dark taxa and parallel taxonomic classifications. Nucleic Acids Res.

[52] Team, R. A language and environment for statistical computing. Computing, 2006. **1**.

[53] Silverman, JD., et al. A phylogenetic transform enhances analysis of compositional microbiota data. Elife, 2017; 6. 10.7554/eLife.21887 PMC5328592 28198697

[54] Dixon, P. VEGAN, a package of R functions for community ecology. J Veg Sci. 2003. 14(6): p. 927–930.

[55] McMurdie PJ, Holmes S. Phyloseq: an R package for reproducible interactive analysis and graphics of microbiome census data. PLoS One. 2013; 8(4): p. e61217. 10.1371/journal.pone.0061217 PMC3632530 23630581

[56] Richards VP, et al. Microbiomes of site-specific dental plaques from children with different caries status. Infect Immun, 2017. 85(8). 10.1128/IAI.00106-17 PMC5520424 28507066

[57] Dixon P (2003). VEGAN, a package of R functions for community ecology. J Veg Sci.

[58] Griffen AL (2019). Significant effect of HIV/HAART on oral microbiota using multivariate analysis. Sci Rep.

[59] Pinto-Cardoso S, Klatt NR, Reyes-Teran G (2018). Impact of antiretroviral drugs on the microbiome: unknown answers to important questions. Curr Opin HIV AIDS.

