# Supplementary material for: Dental caries and its association with the oral microbiomes and HIV in young children—Nigeria (DOMHaIN): a cohort study
Source: BMC Oral Health. 2021 Dec 4;21:620. doi: 10.1186/s12903-021-01944-y (PMC8642767; doi:10.1186/s12903-021-01944-y)
Supplement: Supplementary file 1 — Additional file 1. Supplementary Methods for HIV testing and status determination: Study Procedures. [file 12903_2021_1944_MOESM1_ESM.docx]

**SUPPLEMENTARY METHODS**

**Determine^TM^HIV1/2 Assay Kit**

**Principle**

The Abbot Determine^TM^ HIV 1/2 is an immuno-chromatographic test used in this study as first line of HIV screening kit for the qualitative detection of antibodies to HIV-1/2 in human plasma. The test is intended as an aid to detect antibodies to HIV1/2 from infected individuals. Sample added to the sample pad, migrates through the conjugate pad, it reconstitutes and mixes with the selenium colloid-antigen conjugate. This mixture continues to migrate through the solid phase to the immobilized recombinant antigens and synthetic peptides at the patient window site. If antibodies to HIV-1 and/or HIV-2 are present in the sample, the antibodies bind to the antigen-selenium colloid and to the antigen at the patient window, forming a red line at the patient window site. If antibodies to HIV-1 and/or HIV-2 are absent, the antigen-selenium colloid flows past the patient window and no red line are formed at the patient window site [41].

**Procedure**

The sample used for the serology determination HIV in this study was plasma. The whole blood collected was centrifuged at 4000 rpm for 5mins and 50 µl of the plasma sample with the aid of precision pipette was applied to the sample pad of the determine HIV kit (marked by the arrow symbol). Sample was allowed to flow through capillary action and react for a minimum of 15 minutes (up to 30 minutes) and results was read.

**Uni-GOLD^TM^**

**Principle**

The Uni-gold HIV screening kit was used in this study to confirm positive results from Determine^TM^ HIV kit. Recombinant proteins representing the Immunodominant regions of the envelope proteins of HIV-1 and HIV-2, glycoprotein gp41, gp 120 (HIV-1) and glycoprotein gp36 (HIV-2) respectively are immobilized at the test region of the nitrocellulose strip. These proteins are also linked to colloidal gold and impregnated below the test region of the device. A narrow band of the nitrocellulose membrane is also sensitized as a control region. During testing two drops of serum, plasma or whole blood is applied to the sample port, followed by two drops of wash buffer and allowed to react. The antibody protein-colloidal gold complex moves chromatographically along the membrane to the test and control regions of the test device. A positive reaction is visualized by a pink/red band in the test region of the device. A negative reaction occurs in the absence of human immunoglobulin antibodies to HIV in the analyzed specimen. Consequently no visually detectable band develops in the test region of the device.

Excess conjugate forms a second pink/red band in the control region of the device. The appearance of this band indicates proper performance of the reagents in the kit.

**Procedure**

The required number of Trinity Biotech Uni-Gold^TM^ HIV test devices was removed from their protective wrappers and labeled each test kit with the appropriate participants ID.

Using automatic precision pipettes 50ul filled plasma was applied on the sample port of the kit. Two drops of the buffer was applied and allowed to run for 10 minutes and results were read [41].

**Chembio HIV 1/2 STAT-PAK^TM^ assay.**

**Principle**

The Chembio HIV 1/2 STAT-PAK^TM^ used as a tie breaker with there was a discordant result from the two kits mentioned above. Assay employs a unique combination of a specific antibody binding protein, which is conjugated to colloidal gold dye particles and HIV-1/2 antigens which are bound to the membrane solid phase. The sample is applied to the Samples (S) well followed by the addition of running buffer. The buffer facilitates the lateral flow of the released products and promotes the binding of antibodies to the antigens. If present the antibody binds to the gold conjugated antibody binding protein. In a reactive sample, the dye-conjugated immune complex migrates on the nitrocellulose membrane and is captured by the antigens immobilized in the Test (T) area producing a pink/purple line. In the absence of antibodies, there is no pink/purple line in the T area. The sample continues to migrate along the membrane and produce a pink/purple line in the Control (C) area containing Immuno-globin G antigens. This procedural control serves to demonstrate that specimen and reagents have been properly applied and have migrated through the device. Whole blood (finger stick or venous), serum and plasma may be used. Patients’ samples perform best when treated immediately after collection. If not tested immediately, specimens should be refrigerated at 2-8 ^o^C (36 to 46^o^F) and can be used 3 days after collection. If testing is not possible within 3 days, serum and plasma specimens should be stored frozen at -20^o^C (-4^o^F) or colder. The Immuno-chromatographic rapid qualitative screening test for the detection of antibodies to HIV-1/2 in human finger stick and venous whole blood, serum and plasma is a single-use. The Chembio HIV 1/2 STAT-PAK^TM^ assay is intended for use as a point of care test to aid in the diagnosis of infection with HIV- 1 and HIV- 2. This test is suitable for use in multi-test algorithms designed for the statistical validation of rapid HIV test results. When multiple rapid HIV tests are available, this test should be used in appropriate multi-test algorithms.

### **Procedure**

### Chembio HIV1/2 STAT-PAK^TM^ test device was removed from its pouch and placed on a flat surface (it is not necessary to remove the desiccant from the pouch) and labeled. The 5μl loop provided was dipped into the plasma specimen and transferred to the sample pad. Three drops of buffer was added and allowed to react for 20 minutes and result read [41].

**Determination of EID with dry blood spot using The COBAS® AmpliPrep/COBAS®** **TaqMan® HIV-1.**

**Principle**

The COBAS® AmpliPrep/COBAS® TaqMan® HIV-1 Qual Test is a qualitative test for the detection of Human Immunodeficiency Virus Type 1 (HIV-1) RNA and proviral DNA in plasma, anticoagulated fresh whole blood and dried blood spot (DBS). This test uses the COBAS® Ampliprep Instrument for automated sample processing and the COBAS® TaqMan Analyzer or COBAS® TaqMan® 48 Analyzer for automated amplification and detection. The COBAS® AmpliPrep/COBAS® TaqMan® HIV-1 Test, v2.0 is based on three major processes: (1) sample pre- extraction and incubation, (2) sample preparation to isolate HIV-1 target nucleic acids; (3) reverse transcription of target RNA to generate complementary DNA (cDNA), and (4) simultaneous amplification of target cDNA or proviral DNA by Polymerase Chain Reaction (PCR) and detection of cleaved dual-labeled oligonucleotide detection probe specific to the target. The COBAS® AmpliPrep/COBAS® TaqMan® HIV-1 Qual Test is used with EDTA plasma, fresh EDTA anti-coagulated whole blood and dried blood spot (DBS) punches as sample types. These sample types require a manual, pre-analytical sample extraction. This step is executed using a separate reagent kit, the Specimen Pre-Extraction Reagent (SPEX), consisting of a lysis reagent. The COBAS® AmpliPrep/COBAS® TaqMan® HIV-1 Qual Test permits automated sample preparation followed by automated reverse transcription, PCR amplification and detection of HIV-1 target RNA or proviral DNA and HIV-1 Internal Control (IC) Armored RNA. The Master Mix reagent contains primers and probes specific for both HIV-1 target RNA or proviral DNA and HIV-1 IC RNA. The detection of amplified DNA is performed using target-specific and IC-specific dual-labeled oligonucleotide probes that permit independent identification of HIV-1 target amplicon and HIV-1 IC amplicon [60, 61].

**Procedure**

Input S-tubes were labeled and opened in a safety hood, and the opened Input S-tubes were placed in position 3 through 24 on the Effendorf Thermomixer IsoRack or Sarstedt tube rack as needed, position 3 and 4 were for the CDC negative and CDC positive controls respectively. 12mm circular punches from Whatmann 903 filter cards was excised by manual cutting with sterile scissors by following the circular outline on the Whatmann 903 filter card.

The excised blood spot was transferred into the Input S-tube, which was achieved by folding the spot along its diameter and carefully placed in the middle of the tube aseptically avoiding contamination. Under clean conditions, 1100µl of SPEX was added to each Input S-tube individually and immediately the Input S-tube was closed with the cap. 1000µl of the vortex mixed CTM (-) C (Negative control) was added to all Input S-tube and placed in position 1 of the Effendorf Thermomixer IsoRack and recap. 1000µl of the vortex mixed CTM (-) C (positive control) was added to all Input S-tube and placed in position 2 of the Effendorf Thermomixer IsoRack and recapped

| **SK24 Rack Positions** | |
| --- | --- |
| Sample | Input S-tube |
| **CTM (-) C** | 1 |
| **HIV-1 L (+) C** | 2 |
| Blood spot filter paper punch+ 1100µL **SPEX** | 3-24 |

24 Input S-tubes filled with the samples and controls were incubated in an Effendorf Thermomixer Comfort at 56°C and 1000rpm continuous shaking for 10 minutes.

During the incubation time, an SK24 rack was prepared as follows: Barcode label clip was attached to each SK24 rack position where a sample Input S-tube is to be placed. Note: - The CDC negative and positive controls were treated as samples.

Samples and control orders for the SK24 rack on the AMPLILINK data station were created, and the AMPLILINK software was used to create specimen order for each specimen and control in the orders window sample folder. Immediately after completion of the incubation step, all Input S-tubes were removed and transferred one by one from the Effendorf Thermomixer IsoRack to the SK24, each Input S-tube was removed from the thermomixer IsoRack and placed in the corresponding position on the SK24 rack.

| **SK24 Rack Positions** | | |
| --- | --- | --- |
| Sample | Thermomixer IsoRack | SK24 Rack |
| CTM (-) C | 1 | 1 |
| HIV-1 L (+) C | 2 | 2 |
| CDC negative & positive DBS (Punch  + 1100µl SPEX | 3-4 | 3-4 |
| Sample Punch + 1100µL SPEX | 5-24 | 5-24 |

SK24 rack(s) with Input S-tubes and K-tubes (one for each Input S-tubes, loaded in the right position adjacent to Input S-tubes) was loaded onto rack positions **F, G** or **H** of the COBAS® AmpliPrep Instrument.

Samples and controls were removed from the COBAS® AmpliPrep Instrument on K- carrier racks. Thermal cycler cover was opened and K-carriers were manually transferred into thermal cycler using the K-carrier transporter and lid closed before the COBAS® TaqMan® 48 Analyzer run started. The COBAS® TaqMan® 48 Analyzer run started within 120 minutes following completion of sample and control preparation. At the completion of the COBAS® TaqMan® 48 Analyzer run, results were printed and reported [60-62].

**Results**

The COBAS® TaqMan® 48 Analyzer automatically determines whether HIV-1 RNA or proviral DNA and HIV-1 IC RNA are detected for the sample control.

**AMPLILINK Software**

Amplilink software was used to determine the Cycle threshold value (**Ct**) for the HIV-1 RNA or proviral DNA and the HIV-1 IC RNA. The determined HIV-1 RNA or proviral DNA was detected based upon the Ct values and the fluorescence intensities for the HIV-1 RNA or proviral DNA and HIV-1 IC RNA. The determined HIV-1 L (+) C and CTM (-) C were valid.

References

1. Charles T, E.O., *Human immunodeficiency virus testing algorithm in resource limiting settings*, in *Current perspectives in HIV infection [Internet]*, S. Saxena, Editor. 2013: InTech.

2. Pyne, M.T., K.L. Brown, and D.R. Hillyard, *Evaluation of the Roche Cobas AmpliPrep/Cobas TaqMan HIV-1 test and identification of rare polymorphisms potentially affecting assay performance.* J Clin Microbiol, 2010. **48**(8): p. 2852-8.

3. Tung, Y.C., et al., *Comparison of the Roche COBAS AmpliPrep/COBAS TaqMan HIV-1 test v1.0 with v2.0 in HIV-1 viral load quantification.* Kaohsiung J Med Sci, 2015. **31**(4): p. 188-93.

4. Shin, K.H., et al., *Comparison of the cobas Human Immunodeficiency Virus 1 (HIV-1) Test Using the cobas 4800 System With COBAS AmpliPrep/COBAS TaqMan HIV-1 Test and Abbott RealTime HIV-1 Assay and Performance Evaluation of cobas HIV-1.* Am J Clin Pathol, 2019. **152**(5): p. 558-562.
